# Diverse habitat use during two life stages of the critically endangered Bahama Oriole (*Icterus northropi*): community structure, foraging, and social interactions

**DOI:** 10.7717/peerj.3500

**Published:** 2017-06-22

**Authors:** Melissa R. Price, William K. Hayes

**Affiliations:** 1 Department of Natural Resources and Environmental Management, University of Hawai‘i at Mānoa, Honolulu, HI, United States of America; 2 Department of Earth and Biological Sciences, Loma Linda University, Loma Linda, CA, United States of America

**Keywords:** Caribbean, Dry tropical forest, Synanthropic species, Anthropogenic habitat, Pine forest, Delayed plumage maturation

## Abstract

Our ability to prevent extinction in declining populations often depends on effective management of habitats that are disturbed through wildfire, logging, agriculture, or development. In these disturbed landscapes, the juxtaposition of multiple habitat types can be especially important to fledglings and young birds, which may leave breeding grounds in human-altered habitat for different habitats nearby that provide increased foraging opportunities, reduced competition, and higher protection from predators. In this study, we evaluated the importance of three habitat types to two life stages of the critically endangered Bahama Oriole (*Icterus northropi*), a synanthropic songbird endemic to Andros, The Bahamas. First, we determined the avian species composition and relative abundance of *I. northropi* among three major vegetation types on Andros: Caribbean pine (*Pinus caribaea*) forest, coppice (broadleaf dry forest), and anthropogenic areas, dominated by nonnative vegetation (farmland and developed land). We then compared the foraging strategies and social interactions of two age classes of adult Bahama Orioles in relation to differential habitat use. Bird surveys late in the Bahama Oriole’s breeding season indicated the number of avian species and Bahama Oriole density were highest in coppice. Some bird species occurring in the coppice and pine forest were never observed in agricultural or residential areas, and may be at risk if human disturbance of pine forest and coppice increases, as is occurring at a rapid pace on Andros. During the breeding season, second-year (SY) adult Bahama Orioles foraged in all vegetation types, whereas after-second-year (ASY) adults were observed foraging only in anthropogenic areas, where the species nested largely in introduced coconut palms (*Cocos nucifera*). Additionally, SY adults foraging in anthropogenic areas were often observed with an ASY adult, suggesting divergent habitat use for younger, unpaired birds. Other aspects of foraging (vegetation features, food-gleaning behavior, and food items) were similar for the two age classes. Older Bahama Orioles exhibited relatively higher rates of social interactions (intraspecific and interspecific pooled) in anthropogenic areas, and won more interaction outcomes compared to younger adults. Our findings concur with those of other studies indicating dry broadleaf forest is vitally important to migrating, wintering, and resident birds, including the critically endangered Bahama Oriole, which appears to depend heavily on this vegetation type during certain life stages.

## Introduction

Conservation of endangered species often depends on effective management within human-modified landscapes (Gardner et al., 2009). Resource subsidies in anthropogenic areas, such as cultivated plants or discarded food items, influence avian distribution, abundance, and productivity (Faeth et al., 2005). Synanthropic species, which affiliate with humans, often increase in disturbed areas (Kamp et al., 2009; Coulombe, Kesler & Gouni, 2011), whereas other species, including many Nearctic-Neotropical migrants, may avoid such areas or decline following disturbance (Miller et al., 2007), particularly where much of the canopy is removed (Norris, Chamberlain & Twedt, 2009).

Some species may be negatively affected by expanding agriculture and development if multiple habitat types are required to sustain viable populations (Cohen & Lindell, 2005). The juxtaposition of multiple habitat types can be especially important to fledglings and young birds, which may leave breeding grounds in human-altered habitat for different habitats nearby that provide increased foraging opportunities and higher protection from predators (Cohen & Lindell, 2004; Ausprey & Rodewald, 2011; Price, Lee & Hayes, 2011). Juveniles are often less efficient foragers than adult birds (Wunderle, 1991; Heise & Moore, 2003; Gall, Hough & Fernández-Juricic, 2013), and may seek habitats with reduced competition from conspecifics. To effectively target conservation efforts, we must therefore understand the relative contribution of each habitat type to each life stage, and by extension, population stability (Dent & Wright, 2009).

The Bahama Oriole (*Icterus northropi*), a critically endangered island endemic extirpated from Abaco Island, the Bahamas in the late 20th century and remaining today only on Andros Island, the Bahamas, has contended with profound habitat changes since the arrival of humankind (Steadman et al., 2015; Steadman & Franklin, 2015). While logging and human development removed native breeding and foraging habitats (Currie et al., 2005), and potentially impacted species sensitive to roads and clearings (Laurance, Goosem & Laurance, 2009), humans provided novel opportunities for feeding and nesting in the form of introduced plant species (Nickrent, Eshbaugh & Wilson, 1988). Coconut palms (*Cocos nucifera*), for example, were imported to the region by humans about 500 years ago (Child, 1974; Baudouin & Lebrun, 2009), and have become the Bahama Oriole’s favored nesting habitat (Allen, 1890; Baltz, 1996; Baltz, 1997), most likely due to a preference for the tallest trees available within a nest-site (Price, Lee & Hayes, 2011).

Although largely a synanthropic species associated with human-altered landscapes during the breeding season (Price, Lee & Hayes, 2011), the Bahama Oriole, like other species (Vega Rivera, Rappole & Haas, 1998; Graham, 2001), may still depend on other vegetation types to sustain various activities throughout its life cycle, and may benefit from foraging in multiple vegetation types (Cohen & Lindell, 2005), including dry tropical forest (coppice), pine forest, and human-altered (anthropogenic) areas. Dry tropical forest comprises one of the most endangered tropical ecosystems globally due to anthropogenic disturbance (Janzen, 1988; Gillespie et al., 2012; Banda et al., 2016). In the Bahamas, dry tropical broadleaf forest (coppice) decreased due to the effects of forest fires on ecological succession, and forest clearing in the mid-1900s, but has largely recovered since then. However, much of what remains lacks protection, and the secondary forest lacks heterogeneity (Myers, Wade & Bergh, 2004; Currie et al., 2005) but may still be an important contributor to avian species diversity (Dent & Wright, 2009). Recently, coppice loss has accelerated due to development, increased frequency of human-caused fires, and invasion by non-native vegetation during succession (Smith & Vankat, 1992; Myers, Wade & Bergh, 2004; Koptur, William & Olive, 2010; Thurston, 2010; Carey et al., 2014, see also Larkin et al., 2012).

Because interspecific and intraspecific interactions can be influenced by habitat distribution and foraging strategies (Mac Nally & Timewell, 2005; Shochat et al., 2010), we needed a better understanding of the avian community structure in habitats used by the Bahama Oriole. Thus, we began by determining the avian species composition and relative abundance of the Bahama Oriole among three major vegetation types on Andros: Caribbean pine forest, coppice, and anthropogenic areas.

Next, we sought to better understand the importance of these three vegetation types to two life stages of the Bahama Oriole. In Bahama Orioles, both males and females in their second year of life, though reproductively viable (Price, Lee & Hayes, 2011), display plumage coloration similar to immature birds, a trait referred to as delayed plumage maturation. This trait, though common in males of many species, is not a common characteristic in females (Bentz & Siefferman, 2013), and may contribute to reduced aggression from older birds (VanderWerf & Freed, 2003), reduced predation risk (Lyu, Lloyd & Sun, 2015), and increased overwinter survival (Berggren, Armstrong & Lewis, 2004). A previous study suggested that pine forests and coppice may be particularly important for young Bahama Orioles (Price, Lee & Hayes, 2011). Thus, in the next phase of our study, we compared the foraging strategies and social interactions (both intraspecific and interspecific) of two age classes of adult Bahama Orioles, second-year adults and after-second-year adults, in relation to differential habitat use.

**Figure 1:**
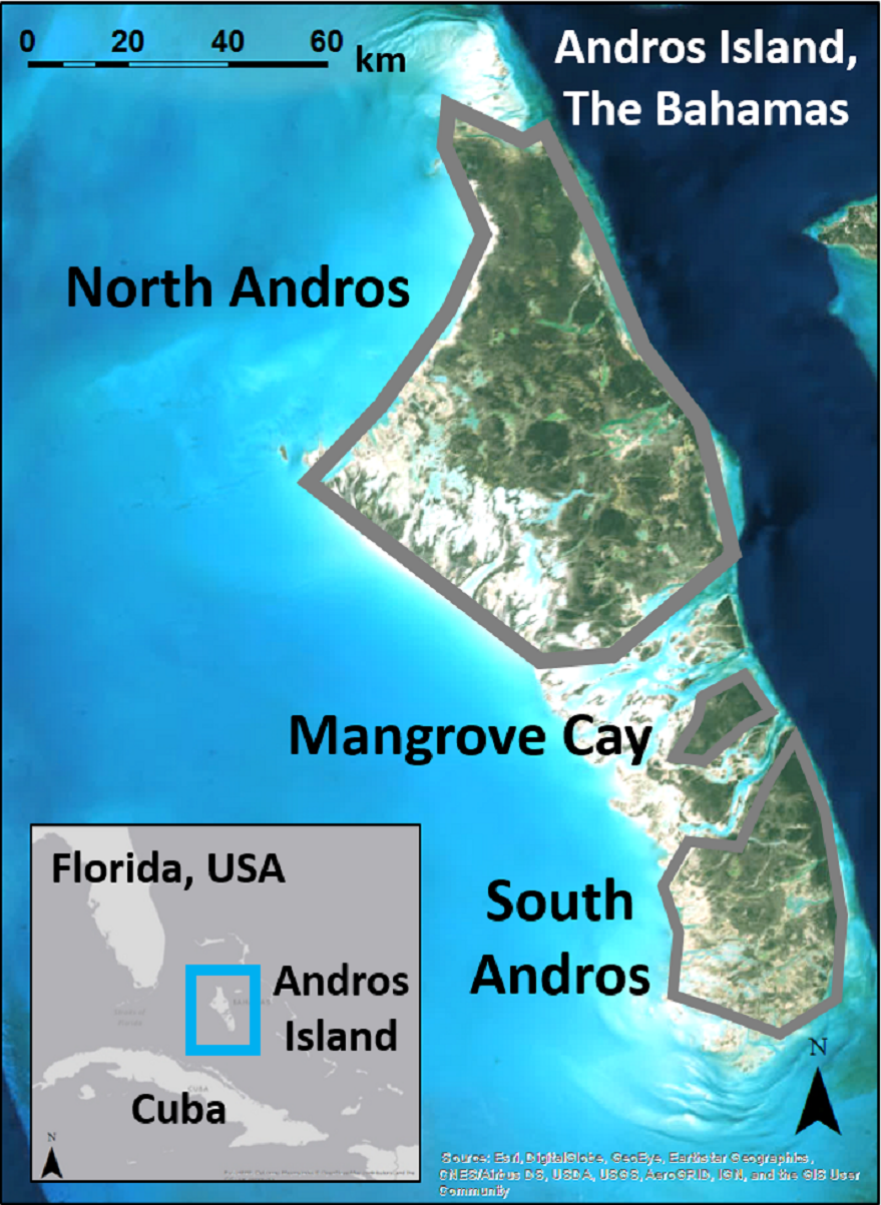
Andros Island is divided into three large, inhabited cays (North Andros, Mangrove Cay, South Andros), and many smaller, uninhabited cays, by channels up to 5 km across. Map data: Esri, Digital Globe, GeoEye, Earthstar Geographics, CNES/Airbus, DS, USDA, USGS, AeroGRID, IGN, GIS User Community.

## Methods

### Study area

Andros Island, The Bahamas, is divided by bights up to 5 km wide into three main human-occupied cays (North Andros, Mangrove Cay, South Andros), and many smaller, uninhabited cays (Fig. 1). Andros Island is dominated on the eastern portion by extensive Caribbean pine forest, with coppice (dry broadleaf forest) at higher elevations and in patches interspersed within the pine forest. Mangrove, associated with vast tidal wetlands and accessible only by boat, dominates the western half. The pine forest was heavilylogged in the mid-1900s (Myers, Wade & Bergh, 2004), and old logging roads provide the only ground access to the interior. Pine trees in the secondary forest are slender and closely spaced, with an understory of poisonwood (*Metopium toxiferum*) and palmetto (*Sabal palmetto*), fern, or shrub (Currie et al., 2005). Human settlements and agricultural developments are spread along the eastern portion of the island.

### Population surveys and observational effort

Other studies have assessed bird composition on Andros during the winter (Currie et al., 2005) and early breeding seasons (Lloyd & Slater, 2010; Price, Lee & Hayes, 2011). To evaluate population density and species composition late in the Bahama Oriole’s breeding season, we conducted line transects between 5–18 July 2005 in coppice, pine forest, and anthropogenic areas on North Andros, using methods similar to Emlen (1971), Emlen (1977) and Hayes et al. (2004). During this time period we expected some Bahama Oriole chicks would have fledged, while others were initiating second broods. We walked individually or with an assistant at approximately 1 km/h, surveying 33 transects totaling 19.5 km, with 13 surveys in coppice (9.8 km), 10 surveys in pine forest (2.4 km), and 10 surveys in anthropogenic areas (7.3 km). Each habitat type was variable, and we sampled across the range of variability. Coppice (dry broadleaf habitat) ranged from 2 m to 10 m in height. Pine forest survey sites included forest with an understory of poisonwood (*Metopium toxiferum*) and palmetto (*Sabal palmetto*), fern, or shrub, and at least one area that had not been previously logged (primary forest). Anthropogenic areas included lands with low-density houses, stores, and other buildings, as well as agricultural, cleared lands with nearby residential dwellings. We recorded all birds identified by sight or vocalization to compare relative and habitat-specific abundance of Bahama Orioles with other species. Research was approved by the Loma Linda University Institutional Animal Care and Use Committee (Protocol 8120010), and conducted under a Bahamas Ministry of the Environment Research Permit.

### Foraging behavior

We recorded foraging and social interaction data from 17 June to 13 July 2007 and 29 March to 30 May 2009. Total time in direct observation of Bahama Orioles was approximately 122 h. To quantify foraging behaviors, we conducted continuous focal observations of individuals for up to 2 h or until the bird flew out of sight. We recorded the first behavior observed after 10-min intervals, or the first behavior after a location change >10 m, whichever came first. Because foraging birds were often only within eyesight for brief periods of time, resulting in single foraging data points for many birds, we included only the first foraging behavior per bird per day in calculations for statistical analyses. We noted age of the bird as second-year (SY) or after-second-year (ASY) based on plumage coloration (Garrido, Wiley & Kirkconnell, 2005), and recorded foraging variables per Remsen & Robinson (1990), including habitat foraged in, location of the bird, substrate fed upon, and food identity. As one cannot distinguish Bahama Orioles males and females by color or behavior, we did not note the sex of the individual. We also noted foliage species in which foraging occurred, and location of the bird in the vegetation, both horizontally (by dividing the tree into visual thirds of inner, middle, outer) and vertically (using a clinometer). Substrates used during foraging were recorded as air, flowers, berries, leaves, twigs, ground, or bark. Foraging tactics were identified as perch gleaning (picking food from a nearby substrate while perched), hang gleaning (picking food from a substrate while hanging upside down), or air-gleaning (plucking insects from the air). We recorded the type of food eaten if it could be identified.

### Social interactions

During the aforementioned focal observations, all intraspecific and interspecific interactions were also noted, per Bowman et al. (1999), as an aerial chase, tree chase, lunge, or usurp. The species and sex (if they could be determined) of the birds were noted, as well as the directionality of each interaction, and outcome of the interaction, such as whether each bird flew away or remained.

### Statistical analyses

We used both parametric and non-parametric tests (Zar, 1996) depending on the nature of the dependent measure and whether or not assumptions were met. We compared the distribution of individual bird species among the three habitats using Kruskal-Wallis ANOVAs. We compared foraging variables and social interactions between SY and ASY adult Bahama Orioles using chi-square tests for categorical data and independent-samples *t*-tests for continuous data, with habitat categories collapsed to “anthropogenic habitat” and “not anthropogenic habitat” and data from 2007 and 2009 combined due to statistical similarity.

We also computed effect sizes, which are largely independent of sample size (in contrast to statistical significance) and more readily compared among different data sets and different studies (Nakagawa & Cuthill, 2007). For Kruskal-Wallis ANOVAs, we calculated eta-squared (*η*^2^) as *χ*^2^∕*N* − 1 (Green & Salkind, 2005), with values of ∼0.01, ∼0.06, and ≥0.14 loosely considered small, medium, and large, respectively (Cohen, 1988). For pairwise comparisons (*t*-tests), we relied on Cohen’s *d* using Hedges’s pooled standard deviation (Nakagawa & Cuthill, 2007), with ∼0.1, ∼0.5, and ≥ 0.8 deemed small, moderate, and large, respectively (Cohen, 1988). For tests of proportions (*χ*^2^), we computed Phi (*φ*) for 2 × 2 and Cramer’s *V* for larger contingency tables, with ∼0.1, ∼0.3, and ≥0.5 considered small, moderate, and large (Cohen, 1988). Following Nakagawa (2004), we chose not to adjust alpha for multiple tests. Although some chi-square tests did not meet assumptions of minimal expected frequencies, the effect sizes corresponded well with and supported the interpretations of significance. All analyses were performed using SPSS 17.0 (2008), with alpha of 0.05. Values are presented as mean ± 1 SE. Rarefaction analyses to determine the likely number of species per vegetation type were performed using EstimateS with 1,000 runs.

**Table 1:** Relative density by habitat (individuals/km) of birds on North Andros, The Bahamas, from 33 line transects during June and July of 2005, with Kruskal–Wallis ANOVA results (Chi-square and *P* values) and eta-squared (*η*^2^) effect sizes.

Species	Pine	Coppice	Anthropogenic	${\chi }_{2}^{2}$	*P*	*η*^2^
	$\overline{X}\pm \mathrm{SE}$	$\overline{X}\pm \mathrm{SE}$	$\overline{X}\pm \mathrm{SE}$			
American Kestrel (*Falco sparverius*)	0.0	0.0	0.9 ± 0.8	3.94	0.14	0.12
Bahama Mockingbird (*Mimus gundlachii*)	1.4 ± 0.8	4.4 ± 2.3	0.0	6.89	**0.032**	0.22
Bahama Oriole (*Icterus northropi*)	0.0	5.6 ± 4.4	1.2 ± 0.6	3.44	0.18	0.11
Bahama Swallow (*Tachycineta cyaneoviridis*)	0.5 ± 0.4	2.0 ± 1.2	1.5 ± 1.1	0.92	0.63	0.03
Bahama Woodstar (*Calliphlox evelynae*)	0.0	0.2 ± 0.2	0.1 ± 0.1	1.74	0.42	0.05
Bahama Yellowthroat (*Geothlypis rostrata*)	0.2 ± 0.2	1.2 ± 0.6	0.0	6.83	**0.033**	0.21
Bananaquit (*Coereba flaveola*)	2.4 ± 0.8	1.1 ± 0.8	2.9 ± 0.9	4.24	0.12	0.13
Black-and-white Warbler^a^ (*Mniotilta varia*)	0.0	0.2 ± 0.2	0.0	1.67	0.44	0.05
Black-faced Grassquit (*Tiaris bicolor*)	15.4 ± 4.6	5.5 ± 2.3	0.3 ± 0.2	13.05	**0.001**	0.41
Blue-gray Gnatcatcher (*Polioptila caerulea*)	10.2 ± 2.5	4.6 ± 2.4	1.1 ± 0.9	10.56	**0.005**	0.33
Black-whiskered Vireo (*Vireo altiloquus*)	8.3 ± 2.6	12.5 ± 4.2	2.5 ± 1.0	3.81	0.15	0.12
Common Ground-Dove (*Columbina passerine*)	2.1 ± 1.1	2.2 ± 1.0	5.4 ± 2.2	2.93	0.23	0.09
Cuban Pewee (*Contopus caribaeus*)	0.6 ± 0.6	0.2 ± 0.1	0.0	2.97	0.23	0.09
Cuban Emerald (*Chlorostilbon ricordii*)	1.3 ± 1.1	5.3 ± 2.6	5.1 ± 1.4	6.48	**0.039**	0.20
Eurasian Collared-Dove^b^ (*Streptopelia decaocto*)	0.8 ± 0.6	1.5 ± 0.9	7.2 ± 3.7	5.59	0.06	0.17
Gray Kingbird (*Tyrannus dominensis*)	2.4 ± 1.7	5.5 ± 4.5	5.0 ± 1.7	6.00	0.050	0.19
Great Lizard-Cuckoo (*Saurothera merlini)*	0.0	0.2 ± 0.1	0.3 ± 0.3	1.53	0.47	0.05
Greater Antillean Bullfinch (*Loxigilla violacea*)	6.0 ± 3.0	0.4 ± 0.4	0.0	8.20	**0.017**	0.26
Hairy Woodpecker (*Picoides villosus*)	4.2 ± 2.0	3.2 ± 1.6	0.1 ± 0.1	5.74	0.06	0.18
House Sparrow^b^ (*Passer domesticus*)	0.0	1.7 ± 1.7	0.5 ± 0.5	0.81	0.67	0.03
Key West Quail-Dove (*Geotrygon chrysie*)	0.0	0.8 ± 0.8	0.0	3.44	0.18	0.11
Killdeer (*Charadrius vociferous*)	0.0	0.0	1.1 ± 0.9	3.94	0.14	0.12
La Sagra’s Flycatcher (*Myiarchus sagrae*)	0.8 ± 0.7	1.4 ± 0.7	0.0	4.09	0.13	0.13
Laughing Gull (*Leucophaeus atricilla*)	0.0	1.3 ± 1.1	3.0 ± 1.0	10.17	**0.006**	0.32
Loggerhead Kingbird (*Tyrannus caudifasciatus*)	0.0	0.0	0.0	0.00	1.00	0.00
Mangrove Cuckoo (*Coccyzus minor*)	0.0	1.1 ± 1.1	0.0	1.67	0.44	0.05
Northern Bobwhite^b^ (*Colinus virginianus*)	2.3 ± 1.3	0.4 ± 0.3	0.0	4.35	0.11	0.14
Northern Mockingbird (*Mimus polyglottos*)	0.9 ± 0.7	2.2 ± 2.2	7.3 ± 1.8	15.34	**0.001**	0.48
Pine Warbler (*Setophaga pinus*)	2.6 ± 1.1	0.5 ± 0.4	0.0	6.88	**0.032**	0.22
Red-legged Thrush (*Turdus plumbeus*)	1.2 ± 0.6	1.9 ± 0.8	0.0	5.76	0.06	0.18
Red-winged Blackbird (*Agelaius phoeniceus*)	0.0	1.7 ± 1.7	0.1 ± 0.1	0.81	0.67	0.03
Rock Pigeon^b^ (*Columba livia*)	0.2 ± 0.2	0.3 ± 0.3	2.2 ± 2.1	0.50	0.78	0.02
Shiny Cowbird (*Molothrus bonariensis*)	0.2 ± 0.2	0.0	0.3 ± 0.2	3.69	0.16	0.12
Smooth-billed Ani (*Crotophaga ani*)	0.0	0.7 ± 0.5	8.0 ± 6.3	8.08	**0.018**	0.25
Thick-billed Vireo (*Vireo crassirostris*)	0.3 ± 0.3	6.4 ± 1.7	2.5 ± 0.8	11.68	**0.003**	0.37
Turkey Vulture (*Cathartes aura*)	0.0	3.4 ± 2.4	5.4 ± 1.5	10.20	**0.006**	0.32
Western Spindalis (*Spindalis zena*)	7.0 ± 1.7	3.3 ± 1.3	0.3 ± 0.3	11.22	**0.004**	0.35
White-crowned Pigeon (*Patagioenas leucocephala*)	5.0 ± 2.5	5.5 ± 4.4	0.3 ± 0.2	4.46	0.11	0.14
Zenaida Dove (*Zenaida aurita*)	0.0	1.1 ± 1.1	0.0	1.67	0.44	0.05

**Notes.**

a Non-resident migratory species.

b Introduced species.

## Results

### Population densities

The number of avian species detected late in the reproductive season (July) was higher in coppice (34), and roughly equivalent in pine forest (24) and anthropogenic habitat (26) (Table 1). The potential number of species in each habitat, at the 95% confidence interval, was calculated as 35–52 in coppice, 27–40 in pine forest, and 26–40 in anthropogenic habitat. Some species clearly associated with one or two habitats, whereas others were generalists; however, transects with zero counts limited statistical power and our ability to identify possible habitat preferences for a number of species, including the Bahama Oriole (*P* = 0.18, *η*^2^ = 0.11; note moderately large effect size). ASY and SY Bahama Orioles were most numerous in coppice (5.6/km), followed by anthropogenichabitat (1.2/km). Although Bahama Orioles were not detected in pine forest during these surveys, they were occasionally observed in this habitat during subsequent work (Price, Lee & Hayes, 2011).

The density of the Bahama Oriole can be compared to that of other species in Table 1, where some structuring of bird communities is evident. Thick-Billed Vireo (*Vireo crassirostris*) was significantly associated with coppice. Black-Faced Grassquit (*Tiaris bicolor*), Blue-Gray Gnatcatcher (*Polioptila caerulea*), Greater Antillean Bullfinch (*Loxigilla violacea*), Pine Warbler (*Setophaga pinus*), and Western Spindalis (*Spindalis zena*) were significantly associated with pine forest. Cuban Emerald (*Chlorostilbon ricordii*), Eurasian Collared-Dove (*Streptopelia decaocto*), Gray Kingbird (*Tyrannus dominensis*), Laughing Gull (*Leucophaeus atricilla*), Northern Mockingbird (*Mimus polyglottos*), Smooth-Billed Ani (*Crotophaga ani*), and Turkey Vulture (*Cathartes aura*) were significantly more likely to be found in anthropogenic habitat. Bananaquit (*Coereba flaveola*) was significantly more likely to be found in pine forest and anthropogenic habitat.

### Bahama oriole foraging

Of the foraging variables listed in Table 2, only the habitat type in which individuals foraged differed significantly between SY and ASY adults (*P* = 0.003, Φ = 0.58). Whereas SY adults (*N* = 15) foraged in coppice, pine forest, and anthropogenic habitat, ASY adults (*N* = 12) were observed foraging only in anthropogenic habitat. Those SY adults foraging in anthropogenic habitat were often paired with an ASY adult (six of seven individuals). Two additional variables showed moderately large effect sizes (Table 2), suggesting that ASY adults are more general in foraging location and in substrate use, whereas SY birds are more likely to forage near the middle of vegetation (*P* = 0.13, *V* = 0.40) from leaves, twigs, or bark (*P* = 0.14, *V* = 0.45). Most food was obtained through perch-gleaning (93% of 27 observations), on leaves and twigs (60% of 27 observations) in the middle of a branch (46% of 27 observations). Both SY and ASY adults were observed air-gleaning and hang-gleaning, although not all of these observations were included in statistical analysis due to non-independence of data points. Birds were observed eating insects (89% of 27 observations) and berries (11%). Other food items included a Caribbean hermit crab (*Coenobita clypeata*), which a SY bird unsuccessfully attempted to ingest, and an endemic brown anole (*Norops sagrei*), which was fed to hatchlings (Price, Lee & Hayes, 2011). Although Bahama Orioles foraged among flowers and may have ingested nectar, we could not ascertain whether their target was the nectar or insects among the flowers.

**Table 2:** Comparisons of foraging variables between second-year (SY) and after-second-year (ASY) Bahama Oriole (*Icterus northropi*) adults, with chi-square and *t*-test results. Anthropogenic habitat was defined as human-modified areas with buildings nearby, such as residential or agricultural areas. Non-anthropogenic areas included coppice (broad-leaf dry forest) and pine forest.

Foraging variable	SY	ASY	Test statistic (df)	*P*	Effect size^a^
Habitat					
Anthropogenic	*N* = 7	*N* = 12	${\chi }_{1}^{2}=9.10$	**0.003**	Φ = 0.58
Not anthropogenic	*N* = 8	*N* = 0			
Height ($\overline{X} \pm \mathrm{SE}$)	5.2 ± 0.4	4.9 ± 0.9	*t*_25_ = 0.32	0.75	*d* = 0.19
Horizontal location					
Inner	*N* = 3	*N* = 5			
Middle	*N* = 9	*N* = 3	${\chi }_{2}^{2}=4.04$	0.13	*V* = 0.40
Outer	*N* = 2	*N* = 4			
Substrate					
Air	*N* = 1	*N* = 0	${\chi }_{3}^{2}=5.48$	0.14	*V* = 0.45
Berries	*N* = 0	*N* = 2			
Flowers	*N* = 3	*N* = 5			
Leaves, twigs or bark	*N* = 11	*N* = 5			
Behavior					
Air-gleaning	*N* = 1	*N* = 0	${\chi }_{2}^{2}=1.73$	0.42	*V* = 0.25
Hang-gleaning	*N* = 1	*N* = 0			
Perch-gleaning	*N* = 13	*N* = 12			
Food item					
Berries	*N* = 1	*N* = 2	${\chi }_{1}^{2}=0.68$	0.41	Φ = 0.16
Insects	*N* = *14*	*N* = 10			

**Notes.**

a Effect sizes: Phi (Φ), Cohen’s *d*, and Cramer’s *V*; see ‘Methods’.

### Social interactions

Intraspecific and interspecific interactions were rare, with only 15 social interactions witnessed (0.12/h of direct Bahama Oriole observation; Table 3), limiting statistical power. While no comparisons were statistically significant, the large effect sizes suggested that social interactions were more likely to occur in anthropogenic habitat for ASY birds and in other habitats (pine or coppice) for SY birds (*P* = 0.077, *V* = 0.53; note large effect size). Older (ASY) Bahama Orioles were also more likely to “win” altercations than SY birds (*P* = 0.077, *V* = 0.53). The avian species that Bahama Orioles interacted with (intraspecific vs. interspecific; *P* = 0.74, *V* = 0.08) and the approximate height above ground of the interaction (*P* = 0.67, *d* = 0.11) were similar for SY and ASY birds, with small effect sizes.

**Table 3:** Comparisons of social interactions and their outcomes between second-year (SY) and after-second-year (ASY) Bahama Oriole (*Icterus northropi*) adults, with Chi-square and *t*-test results.

Variable	SY	ASY	Test statistic (df)	*P*	Effect size^a^
Habitat^b^^,^^c^					
Anthropogenic	*N* = 1	*N* = 9			
Coppice	*N* = 2	*N* = 1	${\chi }_{1}^{2}=4.26$	0.077	*V* = 0.53
Pine Forest	*N* = 1	*N* = 1			
Outcome^b^					
Oriole won	*N* = 0	*N* = 7	${\chi }_{1}^{2}=4.77$	0.77	Φ = 0.56
Oriole lost	*N* = 4	*N* = 4			
Species^d^					
Bahama oriole	*N* = 1	*N* = 2	${\chi }_{1}^{2}=0.09$	0.74	*V* = 0.08
Northern Mockingbird	*N* = 1	*N* = 3			
La Sagra’s flycatcher	*N* = 0	*N* = 1			
Red-legged thrush	*N* = 1	*N* = 0			
Red-tailed hawk	*N* = 0	*N* = 1			
Shiny cowbird	*N* = 0	*N* = 1			
House sparrow	*N* = 0	*N* = 1			
Smooth-Billed Ani	*N* = 0	*N* = 1			
Yellow-crowned	*N* = 1	*N* = 1			
Night-Heron					
Height above ground ($\overline{X}\pm \mathrm{SE}$)	9.2 ± 2.3	8.8 ± 0.9	*t*_13_ = 0.17	0.67	*d* = 0.11

**Notes.**

a Effect sizes: Phi (Φ), Cohen’s *d*, and Cramer’s *V*; see ‘Methods’.

b Intraspecific and interspecific interactions were pooled for analyses.

c Habitat type was collapsed to “anthropogenic” and “not anthropogenic” for analysis.

d Species was collapsed to “intraspecific” (oriole) and “interspecific” (non-oriole) for analysis.

Intraspecific competitive interactions between Bahama Orioles were not frequent (26.7% of 15 total interactions, and only 0.03/h of direct Bahama Oriole observation). In 2009, at the Atlantic Undersea Test and Evaluation Center (AUTEC) where the highest density of Bahama Orioles on North Andros was observed, two pairs of Bahama Orioles nested within 200 m of one another. One oriole from each pair engaged in an aerial chase at the presumed territory boundary. No physical contact was made, although the orioles sang from their respective territories for approximately 30 min following the encounter. On two other occasions, near the beginning of the nesting season, ASY adult Bahama Oriole pairs were observed chasing SY adults, sometimes tussling with them to the ground.

Several interspecific interactions were observed. Orioles engaged a LaSagra’s Flycatcher (*Myiarchus sagrae*), a Smooth-Billed Ani, a Red-Legged Thrush (*Turdus plumbeus*), and a House Sparrow (*Passer domesticus*) pair when these birds independently flew into a Bahama Oriole nest tree. All were chased away except for the House Sparrow pair, which shared a nest tree with a Bahama Oriole pair. Orioles chased a Shiny Cowbird (*Molothrus bonariensis*) away from their nest area. When foraging on one occasion, Bahama Orioles did not interact with nearby cowbirds. Northern Mockingbirds with a nest nearby chased away Bahama Orioles that strayed too close.

We observed several cooperative efforts to chase away potential predators. On one occasion, a Bahama Oriole and three unidentified passerines chased a Red-tailed Hawk (*Buteo jamaicensis*) from its perch in a Caribbean pine tree. On another occasion, one ASY and two SY Bahama Orioles lunged repeatedly at a Yellow-crowned Night Heron (*Nyctanassa violacea*), which only rarely raids nests (Watts, 2011), for over an hour without displacing it. On two occasions, Gray Kingbirds (*Tyrannus dominensis*) whose territories overlapped with Bahama Oriole territories chased away Turkey Vultures (*Cathartes aura*).

## Discussion

### Population densities and estimates

Coppice, pine forest, and anthropogenic habitats contained both habitat-specialists and habitat-generalists (Table 1). As our surveys were conducted during the breeding season for many of the resident species surveyed, the habitat distributions may not represent a complete picture of the habitats important to long-term survival of both juveniles and adults of the resident species. The Bahama Oriole appears to be somewhat of a habitat-generalist, but this becomes apparent only when considering both breeding and non-breeding periods. The Bahama Oriole associates with anthropogenic habitats during the breeding season, as it prefers to nest in the tallest palms available, which are now introduced coconut palms in the vicinity of human residential areas (Baltz, 1996; Baltz, 1997; Price, Lee & Hayes, 2011). The Bahama Oriole likely also benefits from increased foraging opportunities in other cultivated plants, and ready access to adjacent coppice and pine forest foraging grounds. Our survey results late in the breeding season, however, suggest that fledglings with their parents move out of anthropogenic habitats and into coppice habitat shortly after departure from nests. We also observed a high number of SY individuals foraging and interacting socially in coppice during the breeding season. Moreover, during winter surveys, Currie et al. (2005) detected Bahama Orioles only in coppice and agricultural areas, and did not observe them in pine forest lacking a coppice understory. Thus, these studies illustrate the contrasting needs of these birds for anthropogenic habitat, which is important during the breeding season, and coppice, which appears to be important for fledglings, younger birds, and perhaps birds of all ages outside of the breeding season. Unfortunately, coppice is often cleared by humans for agriculture and residential development (Smith & Vankat, 1992), and has been recently decimated in some areas on South Andros (Lloyd & Slater, 2010; Thurston, 2010). This could decrease foraging opportunities and protection for fledging chicks.

Our July surveys found the highest number of avian species in coppice (34 in coppice, 24 in pine forest, and 26 in anthropogenic habitat; however, note the smaller distance surveyed in pine forest habitat than in coppice). The number of species detected in each habitat type was more than reported in previous studies (Currie et al., 2005; Lloyd & Slater, 2010), but likely underestimated the number of species present in each habitat type (95% CI [35–52] in coppice; 27–40 in pine; 26–40 in anthropogenic habitat). Winter survey results on Andros by Currie et al. (2005) similarly detected the highest total number of species in coppice and shrubby field habitats (26–27 in coppice vs. 19–22 species in pine-dominated habitats; anthropogenic habitats were not included in their study). A second study (Lloyd & Slater, 2010) found 22 species in coppice, 28 in pine forests, and 36 in human-modified habitat. However, these surveys were performed April 24–30, and thus detected migratory species. When non-resident species were removed from the comparison, the number of species in each habitat type (15 in coppice, 23 in pine forests, 19 in human-modified habitat) was less than the number detected in our study, particularly in coppice.

A handful of species were significantly more likely to be found in human-disturbed anthropogenic habitat. These included the Cuban Emerald, Eurasian Collared-Dove, Gray Kingbird, Laughing Gull, Northern Mockingbird, Smooth-Billed Ani, and Turkey Vulture. Turkey Vultures, which frequent locations of trash disposal, clearly benefit from human-provided food resources. Other forms of resource subsidies include cultivated fields, imported plants, and fresh water (Faeth et al., 2005). Birds associating with human-disturbed habitats may also be attracted to the open spaces or edges created as land is cleared for development (Hawrot & Niemi, 1996). The frequent occurrence of Laughing Gulls in anthropogenic habitat may be in large part because of the proximity of anthropogenic areas to coastal areas. Several of these birds, including Laughing Gulls and Turkey Vultures, may opportunistically act as predators on newly-fledged Bahama Oriole chicks. Others, such as the Northern Mockingbird, may provide competition for food items.

Some species, including several endemic species, were never observed in agricultural or residential areas, and may be at risk if human disturbance of coppice and pine forest increases. Among the resident species, the Bahama Mockingbird (*Mimus gundlachii*), Bahama Yellowthroat (*Geothlypis rostrata*), Cuban Pewee (*Contopus caribaeus*), Greater Antillean Bullfinch (*Loxigilla violacea*), Key West Quail-Dove (*Geotrygon chrysia*), La Sagra’s Flycatcher, Mangrove Cuckoo (*Coccyzus minor*), and Pine Warbler (*Setophaga pinus*) were never observed in anthropogenic habitat during this study, or during subsequent observations.

### Foraging

Food availability and diet composition of the Bahama Oriole may change throughout the year. One study found protein-rich invertebrates to be the most common food delivered to hatchlings of the closely related Cuban Oriole (*I. melanopsis*), Hispaniolan Oriole (*I. dominicensis*), and Puerto Rican Oriole (*I. portoricensis*), whereas orioles outside of the breeding season more often fed on carbohydrate-rich fruit, flowers, and nectar (Garrido, Wiley & Kirkconnell, 2005). Insects generally have higher densities in pine forest and coppice habitats, whereas fruit and nectar are more abundant in recently disturbed areas (Currie et al., 2005). Thus, we expected breeding Bahama Orioles to forage preferentially in pine forest and coppice, and non-breeding Bahama Orioles to forage more often in anthropogenic habitat. Our breeding season observations of the oriole’s diet composition corresponded with previous studies of related orioles in general composition, as it included fruit, nectar, arthropods, and occasional small vertebrates (Garrido, Wiley & Kirkconnell, 2005). Foraging method was also consistent with expectations, as most food was obtained through perch-gleaning (93% of 27 observations), a simple and relatively inexpensive method in terms of energy (VanderWerf, 1993). Contrary to expectations, however, we found that lone SY orioles foraged only in coppice and pine forest, whereas ASY adults were observed foraging only in anthropogenic habitat. The Bahama Oriole’s proclivity for nesting in coconut palms (Price, Lee & Hayes, 2011), planted primarily in association with human development, may influence the foraging habits of ASY adults, and sufficient protein-rich sources may be present. Interestingly, SY Bahama Orioles paired with an ASY adult almost always foraged in anthropogenic habitat (six of seven observations). Lone SY adults may be forced out of the most desirable habitat due to despotism (Railsback, Stauffer & Harvey, 2003), whereas those paired with older birds, either for breeding or through delayed dispersal, potentially benefit from association with an established territory. Thus, the difference in foraging habitats between SY and ASY adult orioles may have reflected social structure rather than differential food availability among habitats.

### Social interactions

Older (ASY) Bahama Orioles interacted more often with other birds in anthropogenic habitat and “won” altercations more often than younger (SY) Bahama Orioles. The ASY birds in anthropogenic habitat were often nesting or feeding young, and may have had more motivation to defend territories or offspring than SY individuals without a territory to defend. Young adults may also lack the experience to outcompete other birds who challenge them, making it more likely for them to leave an area to avoid more serious altercations.

Intraspecific competitive interactions between Bahama Orioles were rare, probably due to the low density of the Bahama Oriole population. Competitive interactions between ASY adults were only observed in areas where visible signs of lethal yellowing disease were absent from the coconut palms, and Bahama Oriole density was notably higher (c.f., Price, Lee & Hayes, 2011). Aggressive interactions between ASY and SY adults may have involved parents chasing away offspring from a previous brood prior to beginning a new breeding season, or the SY adults may have been young males encroaching on the territories of ASY adults. Long-term studies of individuals marked during their hatch year are needed to elucidate interactions within family groups and during recruitment of juveniles.

### Conservation implications

#### Dry tropical forest

Our findings concur with other studies that indicate coppice is vitally important to resident, migrating, and wintering birds in the Bahamas, including the critically endangered Bahama Oriole (Raffaele et al., 2003; Lloyd & Slater, 2010). Our surveys found the highest number of avian species during the breeding season in coppice (34 in coppice, 24 in pine forest, and 26 in anthropogenic habitat). Young Bahama Orioles often foraged in coppice, and fledglings leaving nests in anthropogenic habitat fledged to coppice (Price, Lee & Hayes, 2011). As all species interact with one or more other species in food webs via competition, predation, parasitism, or mutualism, future studies should elucidate interactions within these habitats, as conservation efforts are more likely to succeed when these complex food web interactions and the ways human activities alter them are understood (Faeth et al., 2005).

#### Caribbean pine forest

Caribbean pine forests on Andros, logged heavily throughout the last century, have returned as homogenous even-aged stands with closely-spaced, slender trees (Currie et al., 2005). This has likely decreased avian diversity compared with old-stand forests, as snags, cavity trees, hardwoods, and large downed woody material are largely absent within secondary-growth pine forests (Thill & Koerth, 2005). Hardwood forests purposefully managed to retain or increase large live trees, snags, and coarse woody debris have increased densities of many birds of conservation concern (Twedt & Somershoe, 2009). Young Bahama Orioles often feed in the pine forest (this study), and adults nest in areas where a palm understory exists (Price, Lee & Hayes, 2011), as well as in suitable pine trees (Stonko et al., in press). Given the importance of the wide swaths of Caribbean pine forest to migratory, wintering, and permanent resident species, conservation plans should consider management of pine forests to increase heterogeneity, and to protect the limited old growth forest that remains.

#### Anthropogenic areas

Not all avian species will decline with human disturbance, and some may even benefit from resource subsidies and increases in open and edge habitats, including those within anthropogenic areas (Werner, Hejl & Brush, 2007; Kamp et al., 2009; Coulombe, Kesler & Gouni, 2011). The Bahama Oriole uses anthropogenic areas during the breeding season, where it selects nest sites in the tallest available palm trees (Price, Lee & Hayes, 2011). Breeding in anthropogenic areas may result in higher levels of nest parasitism from Shiny Cowbirds (Baltz, 1996; Baltz, 1997; Price, Lee & Hayes, 2011), but the benefits of greater nest height for predator avoidance of nonnative cats, rats, and snakes such as the native Bahamian Boa (*Chilabothrus strigilatus*) might offset any such disadvantage (c.f. Burhans & Thompson, 2006). Adult Bahama Orioles in particular appear to benefit from foraging in anthropogenic areas during the breeding season (this study).

The Bahama Oriole requires multiple vegetation types throughout its life history. This is consistent with recent studies which highlight the importance of conserving habitats important to all life stages, rather than only those important to nesting (Streby, Refsnider & Andersen, 2014). In particular, the Bahama Oriole will benefit from careful management of both coppice, which is currently at high risk of rapid loss due to increasing development on Andros (Lloyd & Slater, 2010; Thurston, 2010), and pine forest, which has become more homogenous following deforestation and frequent human-caused forest fires (Currie et al., 2005). Planning for future development on Andros (Inter-American Development Bank, 2014) should make a concerted effort to minimize disturbance of these critical habitats.

## Additional Information and Declarations

### Field Study Permissions

The following information was supplied relating to field study approvals (i.e., approving body and any reference numbers): Field experiments were approved by The Bahamas Environment, Science and Technologies Commission (BEST). No permit number was assigned, but the signed permit is included as supplemental file.

### Data Availability

The following information was supplied regarding data availability: The raw data is included in the tables in the manuscript.

## References

[ref-1] Allen JA (1890). Description of a new species of *Icterus* from Andros Island, Bahamas. Auk.

[ref-2] Ausprey IJ, Rodewald AD (2011). Postfledging survivorship and habitat selection across a rural-to-urban landscape gradient. Auk.

[ref-3] Baltz ME (1996). The distribution and status of the Shiny Cowbird on Andros Island. Bahamas Journal of Science.

[ref-4] Baltz ME (1997). Status of the Black-Cowled Oriole (*Icterus dominicensis northropi*) in the Bahamas. Unpublished report to the Department of Agriculture, Nassau, Bahamas.

[ref-5] Banda K, Delgado-Salinas A, Dexter KG, Linares-Palomino R, Oliveira-Filho A, Prado D, Pullan M, Quintana C, Riina R, Rodriguez GM, Weintritt J, Acevedo-Rodriguez P, Adarve J, Alvarez E, Aranguren AB, Arteaga JC, Aymard G, Castano A, Ceballos-Mago A, Cogollo A, Cuadros H, Delgado F, Devia W, Duenas H, Fajardo L, Fernandez A, Fernandez MA, Franklin J, Freid EH, Galetti LA, Gonto R, Gonzalez-M R, Graveson R, Helmer EH, Idarraga A, Lopez R, Marcano-Vega H, Martinez OG, Maturo HM, McDonald M, McLaren K, Melo O, Mijares F, Mogni V, Molina D, Moreno NDP, Nassar JM, Neves DM, Oakley LJ, Oatham M, Olvera-Luna AR, Pezzini FF, Dominguez OJR, Rios ME, Rivera O, Rodriguez N, Rojas A, Sarkinen T, Sanchez M, Smith C, Vargas B, Villanueva B, Pennington RT (2016). Plant diversity patterns in neotropical dry forests and their conservation implications. Science.

[ref-6] Baudouin L, Lebrun P (2009). Coconut (*Cocos nucifera*) DNA studies support the hypothesis of an ancient Austronesian migration from Southeast Asia to America. Genetic Resources and Crop Evolution.

[ref-7] Bentz AB, Siefferman L (2013). Age-dependent relationship between coloration and reproduction in a species exhibiting delayed plumage maturation in females. Journal of Avian Biology.

[ref-8] Berggren A, Armstrong DP, Lewis RM (2004). Delayed plumage maturation increases overwinter survival in North Island robins. Proceedings of the Royal Society B.

[ref-9] Bowman R, Leonard Jr DL, Backus LK, Mains AR (1999). Interspecific interactions with foraging red-cockaded woodpeckers in South-Central Florida. Wilson Bulletin.

[ref-10] Burhans DE, Thompson FR (2006). Songbird abundance and parasitism differ between urban and rural shrublands. Ecological Applications.

[ref-11] Carey E, Gape BN, Manco D, Hepburn RL, Smith L, D Knowles, Knowles M, Daniels MA, Vincent E, Freid B, Jestrow MP, Griffith M, Calonje AW, Meerow DW, Stevenson J, Francisco-Ortega L (2014). Plant conservation challenges in the Bahama archipelago. Botanical Review.

[ref-12] Child R (1974). Coconuts.

[ref-13] Cohen J (1988). Statistical power analysis for the behavioral sciences.

[ref-14] Cohen EB, Lindell CA (2004). Survival, habitat use, and movements of fledgling White-Throated Robins (*Turdus assimilis*) in a Costa Rican agricultural landscape. Auk.

[ref-15] Cohen EB, Lindell CA (2005). Habitat use of adult White-Throated Robins during the breeding season in a mosaic landscape in Costa Rica. Journal of Field Ornithology.

[ref-16] Coulombe GL, Kesler A, Gouni DC (2011). Agricultural coconut forest as habitat for the critically endangered Tuamotu Kingfisher (*Todiramphus gambieri gertrudae*).

[ref-17] Currie D, Wunderle Jr JM, Ewert DN, Anderson MR, Davis DN, Turner J (2005). Habitat distribution of birds wintering on Central Andros, The Bahamas: implications for management. Caribbean Journal of Science.

[ref-18] Dent DH, Wright JS (2009). The future of tropical species in secondary forests: a quantitative review. Biological Conservation.

[ref-19] Emlen JT (1971). Population densities of birds derived from transect counts. Auk.

[ref-20] Emlen JT (1977). Land bird communities of Grand Bahama Island: the structure and dynamics of an avifauna. Ornithological Monographs.

[ref-21] Faeth SH, Warren E, Shochat PS, Marussich WA (2005). Trophic dynamics in urban communities. BioScience.

[ref-22] Gall MD, Hough LD, Fernández-Juricic E (2013). Age-related characteristics of foraging habitats and foraging behaviors in the black phoebe (*Sayornis nigricans*). Southwestern Naturalis.

[ref-23] Gardner TA, Barlow R, Chazdon RM, Ewers CA, Harvey CA, Peres NS, Sodhi J (2009). Prospects for tropical forest biodiversity in a human-modified world. Ecology Letters.

[ref-24] Garrido OH, Wiley JW, Kirkconnell A (2005). The genus *Icterus* in the West Indies. Neotropical Ornithology.

[ref-25] Gillespie TW, Lipkin L, Sullivan DR, Benowitz S, Pau G, Keppel B (2012). The rarest and least protected forests in biodiversity hotspots. Biodiversity Conservation.

[ref-26] Graham C (2001). Habitat selection and activity budgets of Keel-Billed Toucans at the landscape level. The Condor.

[ref-27] Green SB, Salkind NJ (2005). Using SPSS for Windows and Macintosh: analyzing and understanding data.

[ref-28] Hawrot RY, Niemi GJ (1996). Effects of edge type and patch shape on avian communities in a mixed conifer–northern hardwood forest. Auk.

[ref-29] Hayes WK, Barry Z, McKenzie RX, Barry P (2004). Grand Bahama’s Brown-headed Nuthatch: a distinct and endangered species. Bahamas Journal of Science.

[ref-30] Heise CD, Moore FR (2003). Age-related differences in foraging efficiency, molt, and fat deposition of Gray Catbirds prior to autumn migration. The Condor.

[ref-31] Inter-American Development Bank (2014). BH-T1040: ecosystem-based development for Andros Island. http://idbdocs.iadb.org/wsdocs/getdocument.aspx?docnum=38930099.

[ref-32] Janzen DH, Wilson EO, Peter FM (1988). Tropical dry forest–the most endangered major tropical ecosystem. Biodiversity.

[ref-33] Kamp J, Sheldon MA, Koshkin PF, Donald RD, Biedermann R (2009). Post-Soviet steppe management causes pronounced synanthropy in the globally threatened Sociable Lapwing *Vanellus gregarius*. Ibis.

[ref-34] Koptur S, William P, Olive Z (2010). Ants and plants with extrafloral nectaries in fire successional habitats on Andros (Bahamas). Florida Entomologis.

[ref-35] Larkin CC, Kwit JM, Wunderle EH, Helmer MHH, Stevens MT, Roberts C, Ewert DN (2012). Disturbance type and plant successional communities in Bahamian dry forests. Biotropica.

[ref-36] Laurance WF, Goosem M, Laurance SGW (2009). Impact of roads and linear clearings on tropical forests. Trends in Ecology and Evolution.

[ref-37] Lloyd JD, Slater GL (2010). Rapid ecological assessment of the avian community and their habitats on Andros, The Bahamas. Unpublished report for the Nature Conservancy, Nassau, The Bahamas.

[ref-38] Lyu N, Lloyd H, Sun Y-H (2015). Delayed plumage maturation in birds and the significance of condition-dependent parental care. Behavioral Ecology and Sociobiology.

[ref-39] Mac Nally R, Timewell CAR (2005). Resource availability controls bird-assemblage composition through interspecific aggression. Auk.

[ref-40] Miller C, Niemi JM, Hanowski GJ, Regal RR (2007). Breeding bird communities across an upland disturbance gradient in the Western Lake Superior Region. Journal of Great Lakes Research.

[ref-41] Myers R, Wade C, Bergh D (2004). Fire management assessment of the Caribbean pine (*Pinus caribaea*) forest ecosystems on Andros and Abaco Islands, Bahamas. Global fire initiative 2004–1.

[ref-42] Nakagawa S (2004). A farewell to Bonferroni: the problems of low statistical power and publication bias. Behavioral Ecology.

[ref-43] Nakagawa S, Cuthill IC (2007). Effect size, confidence interval and statistical significance: a practical guide for biologists. Biological Reviews.

[ref-44] Nickrent DL, Eshbaugh WH, Wilson TK (1988). Vascular Flora of Andros Island, Bahamas.

[ref-45] Norris JL, Chamberlain MJ, Twedt DJ (2009). Effects of wildlife forestry on abundance of breeding birds in Bottomland hardwood forests of Louisiana. Journal of Wildlife Managemen.

[ref-46] Price MR, Lee VA, Hayes WK (2011). Population status, habitat dependence, and reproductive ecology of Bahama Orioles: a critically endangered synanthropic species. Journal of Field Ornithology.

[ref-47] Raffaele H, Wiley O, Garrido A, Keith J, Raffaele J (2003). Birds of the West Indies.

[ref-48] Railsback SF, Stauffer HB, Harvey BC (2003). What can habitat preference models tell us? Tests using a virtual trout population. Ecology.

[ref-49] Remsen JV, Robinson SK (1990). A classification scheme for foraging behavior of birds in terrestrial habitats. Studies in Avian Biology.

[ref-50] Shochat E, Lerman JM, Anderies PS, Warren SH, Faeth SB, Nilon CH (2010). Invasion, competition, and biodiversity loss in urban ecosystems. Bioscience.

[ref-51] Smith IK, Vankat JL (1992). Dry evergreen forest (coppice) communities of North Andros Island, Bahamas. Bulletin of the Torrey Botanical Club.

[ref-52] Steadman DW, Albury B, Kakuk JI, Mead JA, Soto-Centeno HM, Singleton NA, Franklin J (2015). Vertebrate community on an ice-age Caribbean Island. Proceedings of the National Academy of Sciences of the United States of America.

[ref-53] Steadman DW, Franklin J (2015). Changes in a West Indian bird community since the late Pleistocene. Biogeography.

[ref-54] Streby HM, Refsnider JM, Andersen DE (2014). Redefining reproductive success in songbirds: moving beyond the nest success paradigm. Auk.

[ref-55] Stonko DC, Rolle LE, Smith LS, Scarselletta AL, Christhilf JL, Rowley MG, Yates SS, Cant-Woodside S, Brace L, Johnson SB, Omland KE New documentation of pine forest nesting by the critically endangered Bahama Oriole (*Icterus northropi*). Journal of Caribbean Ornithology.

[ref-56] Thill RE, Koerth NE (2005). Breeding birds of even- and uneven-aged pine forests of eastern Texas. Southeastern Naturalis.

[ref-57] Thurston G (2010). South Andros farm road progresses. http://www.thebahamasweekly.com/publish/bis-news-updates/South_Andros_farm_road_progresse.

[ref-58] Twedt DJ, Somershoe SG (2009). Bird response to prescribed silvicultural treatments in bottomland hardwood forests. Journal of Wildlife Managemen.

[ref-59] VanderWerf EA (1993). Scales of habitat selection by foraging ‘Elepaio in undisturbed and human-altered forests in Hawaii. The Condor.

[ref-60] VanderWerf EA, Freed LA (2003). ‘Elepaio subadult plumages reduce aggression through graded status-signaling, not mimicry. Journal of Field Ornithology.

[ref-61] Vega Rivera JH, Rappole JH, McShea WJ, Haas CA (1998). Wood Thrush postfledging movements and habitat use in northern Virginia. The Condor.

[ref-62] Watts BD, Rodewald PG Yellow-crowned night-heron (*Nyctanassa violacea*). The birds of North America.

[ref-63] Werner SM, Hejl SJ, Brush T (2007). Breeding ecology of the Altamira Oriole in the lower Rio Grande Valley, Texas. The Condor.

[ref-64] Wunderle JM, Power DM (1991). Age-specific foraging proficiency in birds. Current ornithology.

[ref-65] Zar JH (1996). Biostatistical analysis.

